# Factors associated with patients’ mobility rates within the provinces of Iran

**DOI:** 10.1186/s12913-022-08972-6

**Published:** 2022-12-20

**Authors:** Somayeh Noori Hekmat, Ali Akbar Haghdoost, Zahra Zamaninasab, Rohaneh Rahimisadegh, Fatemeh Dehnavieh, Samira Emadi

**Affiliations:** 1 Health Services Management Research Center, Institute for Futures Studies in Health, Kerman University of Medical Sciences, Kerman, Iran; 2 Health Modeling Research Center, Institute for Futures Studies in Health, Kerman University of Medical Sciences, Kerman, Iran; 3 Department of Biostatistics and Epidemiology, School of Public Health, Kerman University of Medical Sciences, Kerman, Iran; 4 Health Services Management Research Center, Institute for Futures Studies in Health, Kerman University of Medical Sciences, Kerman, Iran; 5 Health Foresight and Innovation Research Center, Institute for Futures Studies in Health, Kerman University of Medical Sciences, Kerman, Iran

**Keywords:** Patient mobility, Specialist distribution, Total health expenditure, Curative services, Hospital

## Abstract

**Background:**

The absence of a referral system and patients’ freedom to choose among service providers in Iran have led to increased patient mobility, which continues to concern health policymakers in the country. This study aimed to determine factors associated with patient mobility rates within the provinces of Iran.

**Methods:**

This cross-sectional study was conducted in Iran. Data on the place of residence of patients admitted to Iranian public hospitals were collected during August 2017 to determine the status of patient mobility within each province. The sample size were 537,786 patients were hospitalized in public hospitals in Iran during August 2017. The patient mobility ratio was calculated for each of Iran’s provinces by producing a patient mobility matrix. Then, a model of factors affecting patient mobility was identified by regression analysis. All the analyses were performed using STATA14 software.

**Results:**

In the study period, 585,681 patients were admitted to public hospitals in Iran, of which 69,692 patients were referred to the hospital from another city and 51,789 of them were admitted to public hospitals in the capital of the province. The highest levels of intra-provincial patient mobility were attributed to southern and eastern provinces, and the lowest levels were observed in the north and west of Iran. Implementation of negative binomial regression indicated that, among the examined parameters, the distribution of specialist physicians and the human development index had the highest impact on intra-provincial patient mobility.

**Conclusion:**

The distribution of specialists throughout different country areas plays a determining role in patient mobility. In many cases, redistributing hospital beds is impossible, but adopting different human resource policies could prevent unnecessary patient mobility through equitable redistribution of specialists among different cities.

**Supplementary Information:**

The online version contains supplementary material available at 10.1186/s12913-022-08972-6.

## Background

Patient mobility is a factor that shows the quality and availability of hospital services in a region [1]. Patients’ mobility from their own region indicates the low quality and quantity of services provided in their region and the unavailability of services [2]. Patient mobility, based on the patient’s preference for one hospital over another, is a negative indicator for understanding the quality and quantity of services provided in the hospital [3]. Inequality in the allocation of resources, especially for access to quality medical services, has an effective role in the intercity mobility of patients. Therefore, patient mobility reflects the unequal distribution of health care resources in different regions [4]. Patient mobility is a significant issue for both patient-receiving and patient-sending areas as it can influence the pattern of health service provision and consumption in both places [5, 6]. Patient mobility is defined as the patient’s mobilization from their place of living to another place, chosen freely by the patient or, occasionally, according to a doctor’s recommendation, to receive medical services [7]. Patient mobility usually occurs due to their freedom to choose a provider [8].

Patient mobility is more common in regions with maldistribution of health service resources and infrastructures [5]. Patient mobility signifies that local health services are poor in terms of equity in access, quality, safety, or costs and is also one of the main reasons for low investment in the development of medical infrastructure in local societies (Local communities mean cities and rural areas in each province) [6]. This phenomenon is known as the death cycle of local hospitals.

Patient mobility is not always considered a negative phenomenon. In some countries, such as the United States and several Western European countries, there are policies that enable the patients to choose freely among health care providers. These policies aim to create competition, increase productivity, and improve the quality of medical care [9, 10]. In fact, in this approach, patient mobility among areas is considered a stimulus to improving the quality of medical care [11].

In Italy, the phenomenon of patients’ mobility has been growing for several years [2]. Factors affecting the increase in patient mobility are:

- a) Patients can receive higher quality health care from specialized supra-regional or international health centers in the context of health tourism. (b) Patients can visit medical centers when they are abroad for other reasons c) Neighboring regions or provinces may have more advanced facilities. (d) due to lack of access to specialized care such as lack of specialist doctors and lack of advanced medical equipment [12].

Patient freedom to choose healthcare providers to spark competition is known as the ‘voting with your feet’ principle [8]. Although patient freedom of choice is one of the regulatory policies in some countries, it is recommended that patient mobility happen within the referral system. In the referral system, health centers and general and specialized hospitals complement each other. Whoever can be treated at a health center or public hospital will be treated there; otherwise, they will be referred to a higher level of referral, usually a specialized or sub-specialized hospital in a larger city. The referral system ensures fair access to services based on patients’ needs [9]. Patient mobility outside the referral system causes overloading of health facilities in patient-receiving areas and non-use of the capacities provided in the patient-sending areas [7]. Moreover, patient mobility creates dissatisfaction and additional social costs, such as travel and accommodation costs, for patients and their companions and prolonged absence from work for citizens who need to receive health care in another area [5].

In Iran, there is a semi-pluralistic health care system, which means the parallel existence of private- and public-owned health facilities and direct and indirect payments for health services. In Iran, there is a robust private health sector delivering curative services. All cities with a population of over fifty thousand have at least one public hospital, whereas almost all private hospitals are located in provincial capitals [7]. Lastly, In Iran’s health system, due to the lack of a mandatory referral system, people can choose the service provider, the level of specialization, and the geographical location to receive the service [13].

Koylu et al. conducted a study in 2018 to analyze province-to-province patient mobility in Turkey from 2009 to 2013. This study used a flow-based regionalization method to discover functional medical regions by studying the patient mobility network. The results emphasized that the medical regions determined by analyzing the patient mobility data showed substantial overlap with the designated regions of the Ministry of Health. Also, it identified several regions where the regional service utilization did not match the planned service delivery [14].

So far, no study has been conducted to measure the rate of patient migration in Iran, and only one study was conducted by Sabermahani with the aim of knowing the reasons for patient migration. Therefore, the present study, for first time in Iran, aimed to estimate the intra-provincial patient mobility ratio and identify the factors affecting it.

## Methods

### Study design

This study was conducted in Iran using a cross-sectional design. Data on patients’ place of residence who were admitted to Iranian public hospitals were collected during August 2017 to determine the status of patient mobility within each province.

### Data collection

The intra-provincial patient mobility ratio was the dependent variable. Patient mobility data were collected using the intra-provincial patient mobility matrices. Iran’s health information system doesn’t gather patient mobility data routinely and we gathered cross-sectional data on patient mobility using a temporary data warehouse that was specially designed for the present study. With the participation of the authorities in medical universities in each province, a patient mobility matrix was compiled for each province.

The header column and the header row of the matrix contained a list of cities in each province. The numbers of patients hospitalized in their city of residence and those who traveled to other cities in the province were recorded using the patients’ admission data. Finally, the intra-provincial mobility ratio was calculated by determining the percentage of patients who left their city to obtain medical treatment in other cities in the province. A total of 31 intra-provincial patient mobility matrices were generated to illustrate intra-provincial patient mobility in each province.

Data were collected from 602 public hospitals, including general, specialized, and sub-specialized hospitals because patients referred to public hospitals are the best representatives of the general public. In addition, due to the insurance coverage of public hospital services, patient admission data in public hospitals are recorded more accurately than in private hospitals. It should also be noted that approximately 80% of hospital beds in Iran are in public hospitals.

In order to eliminate the risk of bias in collecting patient mobility data and considering the normal status of patient admission in summer, when referrals are not affected by seasonal epidemics or road accidents, as they are during autumn and winter or during the spring holidays, patient mobility data were collected during August 2017. Although in August hospitals’ admissions are not affected by seasonal epidemics or air pollution, as they are during autumn and winter [15–17] and elective surgeries may decrease in summer, several studies show summer trips increase the number of road accidents during summer. Considering that August is in the middle of summer, in terms of summer trips, it has a more normal situation than the beginning and end of summer months [18–20].

The sample size was 537,786 patients who were hospitalized in public hospitals in Iran during August 2017. Using the home address record of hospitalized patients, the patient mobility matrix was produced and the patient mobility ratio was calculated for each of Iran’s provinces. Then, a model for factors affecting patient mobility was identified using regression analysis.

Evidence shows that the distribution of health resources and health service accessibility affects patient mobility. For this reason, data on the number and distribution of health resources in different provinces were used to determine factors affecting patient mobility. Independent variables were as follows: Total health expenditure (THE) per capita indicates affordability, under-five mortality rate (U5MR) indicates health service accessibility, population density is a geographical index, and human development index (HDI) is a socio-economic variable that defines province heterogeneity. Definitions of variables and data sources are presented in Table 1.

**Table 1:** Definition and explanation of variables

	**Variable**	**Definition and explanation**	**Data Source**
Dependent variable	Intra-provincial patient mobility rate	The number of patients who left their cities to receive curative services in hospitals in other cities in the province [21] Intra-provincial patient mobility index is calculated as follows: $\frac{\mathrm{total}\;\mathrm{number}\;\mathrm{of}\;\mathrm{patients}\;\mathrm{moving}\;\mathrm{within}\;\mathrm{the}\;\mathrm{province}}{\mathrm{total}\;\mathrm{number}\;\mathrm{of}\;\mathrm{hospitalized}\;\mathrm{patients}\;\mathrm{in}\;\mathrm{the}\;\mathrm{province}}$	Survey using HIS data of governmental hospitals in 349 cities in Iran
Independent variables	Distribution of hospital beds	The number of hospital beds in each city Hospital beds in this study included all inpatient beds used for acute patients in governmental hospitals but did not include long-term care beds	A survey using the NEDA platform^a^ was filled in all medical universities across the country
	Distribution of specialist physicians	The number of specialist physicians in each city A specialist physician is a medical school graduate who has completed advanced training in a specific field of medicine [22]	
	Coefficient of variation of health resources (CV)	The coefficient of variation of health resources indicates the degree of dispersion of resources around the mean. A higher coefficient of variation percentage indicates a greater maldistribution of resources in the country [23]. The index is calculated as follows: $\frac{\mathrm{Standard}\;\mathrm{deviation}}{\mathrm{The}\;\mathrm{average}\;\mathrm{annual}\;\mathrm{number}\;\mathrm{of}\;\mathrm{resources}}$	
	Population density	The number of inhabitants per square kilometer which is calculated by dividing the population by the area of ​​the province [24]	Country Statistics Organization and statistical reports
	Total health expenditure (THE)	Total health expenditures include all health sector expenditures, including costs of treatment, health, prevention, allied health, pharmaceutics, equipment, administration, infrastructure investment, health education, and research. THE is calculated as follows: $\frac{\mathrm{The}\;\mathrm{absolute}\;\mathrm{value}\;\mathrm{of}\;\mathrm{the}\;\mathrm{total}\;\mathrm{health}\;\mathrm{expenditure}}{\mathrm{Population}\;\mathrm{of}\;\mathrm{each}\;\mathrm{province}}$	Total health expenditures and the share of out-of-pocket payments in Iranian provinces [25]
	Human development index (HDI)	Human development index is a composite indicator that shows the impact of economic strategies on human living standards. HDI is calculated by three main factors of income, education, and health [26]	Provincial Human Development Index, a Guide for Efficiency Level Analysis: The Case of Iran [26]
	Under-five mortality rate (U5MR)	Child mortality indices are principal indicators of population health and well-being. Under-five mortality is defined as the probability of a child born in a given year dying before reaching their fifth birthday and is expressed per 1000 live births [27]	Measuring Iran’s success in achieving Millennium Development Goals 4: a systematic analysis of under-5 mortality at national and subnational levels from 1990 to 2015 [28]

^a^The project team developed the NEDA platform to collect data on the number of active hospital beds and medical specialists, the number of patients admitted, and a matrix of patient mobility data in cities affiliated to each University of Medical Sciences.

### Data analysis

In order to analyze the data, the type of response variable must be taken into account. In the present study, as the response variable is the count of the intra-provincial patient mobility, Poisson distribution can be used to analyze the data when the mean distribution of intra-provincial mobility and its variance are equal [29]. However, if overdispersion occurs (when the variance is higher than the mean distribution of intra-provincial mobility), negative binomial distribution must be used. In this study, as overdispersion was observed in the distribution of intra-provincial mobility, negative binomial regression was used.

In the present study, the aforementioned negative binomial regression formula is as follows:

$$\text{ln}\left(E\left(\text{P}\text{M}\right)\right)={\beta }_{0}+{\beta }_{1}\left(HB\right)+{\beta }_{2}\left(Sp\right)+{\beta }_{3}\left(HDI\right)+{\beta }_{4}\left(THE\right)+{\beta }_{5}\left(PD\right)+{\beta }_{6}\left(U5MR\right)$$

where

- PM: patient mobility
- HB: number of hospital beds
- Sp: number of specialist physicians
- HDI: human development index
- THE: total health expenditure
- PD: population density
- U5MR: under-five mortality rate

While the number of health resources indicates health investment status in each province, the coefficient of variation (CV) of health resources indicates their distribution across each province. CV is a standardized measure of frequency distribution defined as the ratio of the standard deviation to the mean [30]. Therefore, to study the effect of the distribution of beds, nurses, and specialist doctors on intra-provincial mobility, the fractional regression model was performed with the “rate” of patient mobility as the response variable. In the end, the *margins* command was used to determine changes in mobility rate brought about by the increase in the coefficient of variation of health resources.

The fractional regression model equation is calculated using the following equation:

$$\text{ln}\left(E\left(\text{P}\text{M}\right)\right)={\beta }_{0}+{\beta }_{1}\left(\text{H}\text{B}\text{C}\text{V}\right)+{\beta }_{2}\left(\text{N}\text{C}\text{V}\right)+{\beta }_{3}\left(\text{S}\text{p}\text{C}\text{V}\right)$$

where

- PM: patient mobility
- HBCV: coefficient of variation of hospital beds
- NCV: coefficient of variation of nurses
- SPCV: coefficient of variation of specialist physicians

**Fig. 1:**
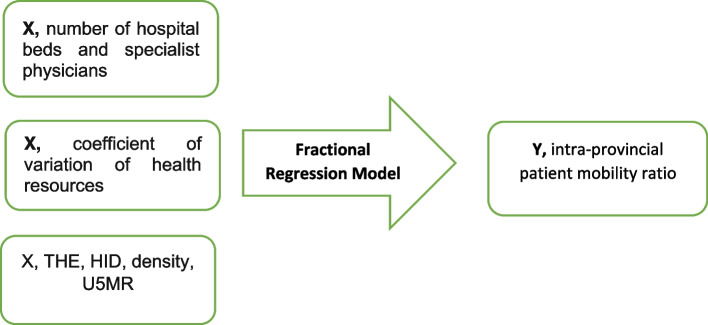
The fractional regression model and independent and response variables

The variables used in the analysis of intra-provincial patient mobility regression (percentage of intra-provincial mobility) include the response variable and the independent variables, including the number of hospital beds and specialist physicians in each province and the coefficient of variation for each of these variables (Fig. 1).

**Fig. 2:**
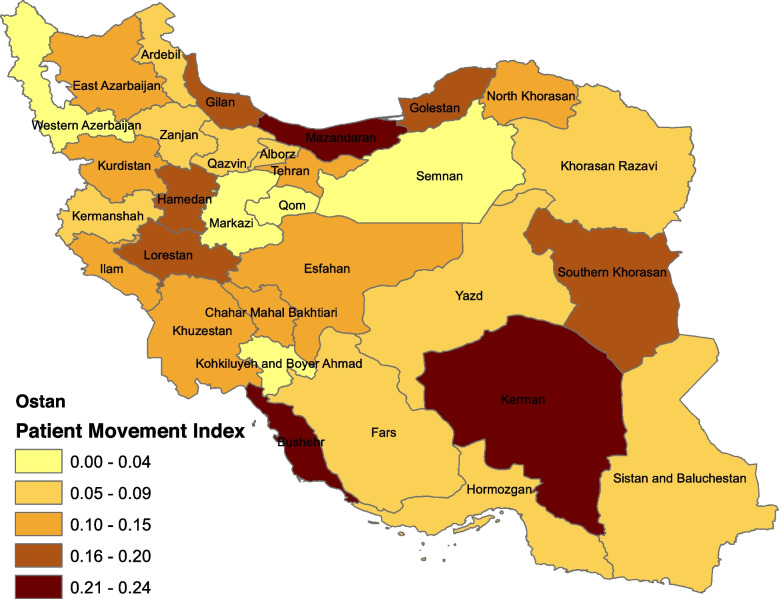
Patient mobility rate in Iran’s provinces

Finally, as the secondary analysis, cluster analysis was performed to group the provinces using the specialist coefficient of variation for each province. In cluster analysis, observations within each cluster are most similar to each other and most different from other clusters. All the analyses were done using STATA 14 software and ArcGIS/ArcMap 10.4.1 software was used to generate Fig. 2. Considering that the range of patient mobility index was between 0 and 0.24, it divided into 5 equal classes.

## Results

### Descriptive findings

The findings of this study show that access to resources and the way they are dispersed varies in different parts of the country (Table 1). There is an average of 1.47 hospital beds, 133 nurses, and 47 doctors per 100,000 people in Iran. Northern provinces of Iran have better access to hospital beds, nurses, and specialists. Furthermore, western provinces are more privileged than eastern ones in this regard. Sistan and Baluchestan, a province located in southeast Iran, is the most deprived province regarding access to hospital beds and specialists.

There is a similar pattern regarding the coefficient of variation of health resources in Iran, with northern provinces showing a lower coefficient of variation of resources than the southern provinces. A lower coefficient of variation of resources shows fair distribution of resources in each province. The highest coefficient of variation of resources was associated with specialist physicians, and the lowest was attributed to hospital beds with coefficients of 30% and 22%, respectively. With a coefficient of variation of 33%, nurses scored almost as low as hospital beds (Table 2).

In the study period (August 2017), 585,681 patients were admitted to public hospitals in Iran, of which 69,692 patients were referred to the hospital from another city and 51,789 of them were admitted to public hospitals in the capital of the province. The highest intra-provincial mobility was observed in the southern and eastern provinces, and the lowest was seen in the northern and western provinces (Table 2). Patient mobility in central areas is lower than in border areas (Fig. 2).

The independent variables’ status in Iran’s provinces is presented in Table 3. As the table shows, the northern and central regions, followed by the western regions, are in a better position to access health resources than the eastern and southern regions.

**Table 2:** Patient mobility rate in Iran’s provinces

**Geographical area**	**Province**	**Population**	**Number of inpatients (August 2017)**	**Number of intra-provincial immigrant patients**	**Share of the capital city of the province**	**Percentage of intra-provincial migration**	**Percentage of migration to the capital city of the province**	**Patient mobility rate**
**North**	Mazandaran	3,171,946	23,261	3,257	1,861	14%	8%	23%
	Gilan	2,533,407	17,312	3,462	1,904	20%	11%	20%
	Golestan	1,896,278	12,168	2,190	243	18%	2%	19%
	East Azarbaijan	3,920,544	29,404	4,705	0	16%	0%	15%
	Ardabil	1,309,768	8,295	498	498	6%	6%	6%
	North Khorasan	927,448	6,338	824	444	13%	7%	13%
**West**	Kohgiloyeh and Boyerahmad	681,147	4,257	85	85	2%	2%	4%
	West Azerbaijan	3,267,165	22,870	915	229	4%	1%	4%
	Kermanshah	2,036,042	15,440	1,235	618	8%	4%	8%
	Kurdistan	1,574,757	9,842	1,083	787	11%	8%	11%
	Hamedan	1,836,489	12,855	2,185	2,185	17%	17%	17%
	Ilam	569,147	3,557	391	391	11%	11%	11%
	Khuzestan	4,843,427	38,344	4,985	4,218	13%	11%	12%
	Lorestan	1,791,285	11,793	2,359	1,887	20%	16%	20%
	Zanjan	1,071,071	8,479	509	170	6%	2%	6%
**South**	Fars	4,823,378	37,783	3,023	1,133	8%	3%	8%
	Hormozgan	1,710,608	11,832	710	355	6%	3%	6%
	Bushehr	1,104,742	6,905	1,519	1,312	22%	19%	22%
**Center**	Tehran	12,707,586	102,720	18,490	18,490	18%	18%	11%
	Qom	1,226,978	8,282	0	0	0%	0%	0%
	Alborz	2,758,741	17,242	1,035	1,035	6%	6%	6%
	Qazvin	1,260,478	8,508	766	681	9%	8%	9%
	Semnan	658,129	4,333	130	87	3%	2%	2%
	Markazi	1,560,281	11,052	332	221	3%	2%	3%
	Esfahan	5,072,296	39,310	4,717	4,324	12%	11%	11%
	Yazd	1,067,793	8,453	507	254	6%	3%	6%
	Chaharmahal and Bakhtiari	929,556	6,197	868	682	14%	11%	14%
**East**	Kerman	3,150,695	22,580	2,484	2,258	11%	10%	24%
	Sistan and Baluchestan	2,634,564	17,783	1,245	889	7%	5%	7%
	Khorasan Razavi	7,043,548	53,414	4,273	3,739	8%	7%	8%
	Southern Khorasan	780,406	5,073	913	812	18%	16%	18%
Total		79,919,699	585,681	69,692	51,789	0.12	0.09	

**Table 3:** The variables’ status in Iran’s provinces

**Geographical area**	**Province**	**Independent variables**							**Coefficient of variation of health resources (CV)**			**Dependent variable**
		**Density**	**U5MR 2014**	**HDI**	**THE per capita**	**Resources Number**						**Patient mobility rate**
						**Hospital bed**	**Nurses**	**Specialists**	**Hospital bed**	**Nurses**	**Specialists**	
**North**	Mazandaran	87	9.33	0.63	7.54	4792	5681	1704	29	23	28	23%
	Gilan	60	16	0.7	8.06	3322	2951	1143	40	33	42	20%
	Golestan	63	17.67	0.65	7.03	2375	2710	677	57	52	56	19%
	East Azarbaijan	33	19	0.66	7.61	5856	5204	1934	32	32	36	15%
	Ardabil	17	19.67	0.7	7.41	1994	1839	426	48	38	41	6%
	North Khorasan	14	32.33	0.62	5.36	1008	1277	233	58	48	55	13%
**West**	Kohgiloyeh and Boyerahmad	2	20.67	0.74	11.79	862	1051	222	63	66	61	4%
	West Azerbaijan	134	27.33	0.72	10.05	4120	3448	920	29	27	36	4%
	Kermanshah	7	23.67	0.74	10.8	2876	2393	557	40	44	41	8%
	Kurdistan	77	32.33	0.72	10.44	2102	2410	492	41	59	49	11%
	Hamedan	14	20.67	0.74	11.85	2660	2196	495	41	43	35	17%
	Ilam	44	13.67	0.65	10.61	811	733	164	47	43	55	11%
	Khuzestan	11	19.67	0.68	11.19	7150	4905	148	54	56	7	12%
	Lorestan	76	16.67	0.73	9.92	2165	1933	465	48	45	52	20%
	Zanjan	39	23	0.74	10.66	1545	1390	394	60	52	67	6%
**South**	Fars	94	27	0.66	13.09	7685	8554	2405	43	38	11	8%
	Hormozgan	57	25.33	0.71	12.42	2084	1636	418	60	67	63	6%
	Bushehr	47	19	0.75	11.88	1337	1209	423	52	60	64	22%
**Center**	Tehran	780	17	0.75	15.92	25,501	20,799	11,226	54	53	15	11%
	Qom	62	13	0.7	8.42	1675	1684	477	0	0	0	0%
	Alborz	54	19.33	0.63	8.46	2125	1829	163	52	53	66	6%
	Qazvin	5	16.67	0.66	8.41	1779	1799	480	67	61	60	9%
	Semnan	73	20	0.65	9.08	1534	1248	356	54	48	16	2%
	Markazi	48	18	0.74	8.67	2054	1576	778	71	44	50	3%
	Esfahan	28	19.67	0.74	9.28	7341	7489	29	46	37	25	11%
	Yazd	81	21.33	0.75	9.08	2538	1952	541	41	49	40	6%
	Chaharmahal and Bakhtiari	24	17.67	0.69	9.6	1442	1385	266	58	58	81	14%
**East**	Kerman	109	21.67	0.72	9.72	4373	4499	1034	56	57	54	24%
	Sistan and Baluchestan	48	28.67	0.67	9.62	2369	1955	419	66	70	13	7%
	Khorasan Razavi	529	26	0.74	9.75	9077	7859	282	61	74	35	8%
	Southern Khorasan	81	47.67	0.66	9.9	1008	871	227	66	50	67	18%
**IR. Iran**						**117,580**	**106,465**	**36,345**	**22**	**23**	**30**	

### Analytical findings

In order to analyze the effect of the availability of health sector resources on intra-provincial patient mobility, negative binomial regression was used. Moreover, the fractional regression model was used to analyze the effect of health sector resource distribution on intra-provincial patient mobility.

Table 4 represents the results of the negative binomial model to determine the effect of the number of hospital beds, the number of specialists, HDI, THE, population density, and U5MR on the number of patients who moved within provinces. The results of the negative binomial model indicate that although the effect of all six variables on the number of intra-provincial mobility was non-significant (*P*-value > 0.05), with a 1000-unit increase in hospital beds, intra-provincial patient mobility increased by 9%. In addition, for each unit increase in U5MR, HDI, and population density, the intra-provincial patient mobility decreased by 1%, 84%, and 1%, respectively. Also, intra-provincial mobility increased by 3% for each unit increase in THE. This model showed that the HDI variable has the highest effect on intra-provincial patient mobility compared to other variables (Table 4).

**Table 4:** Negative binomial model to examine the effect of resource growth in the province on intra-provincial mobility

**Independent Variables**	**Irr**^a^	**SE**	***P*-Value**
Hospital beds	1.09	0.22	0.681
Specialist physicians	0.99	0.53	0.981
Human development index	0.16	0.83	0.722
Total health expenditure	1.03	0.13	0.774
Population density	0.99	0.002	0.293
U5MR	0.99	0.02	0.787

^a^ *irr *This word stands for Incidence Rate Ratio, which is equivalent to Relative Risk

The variables used in the fractional regression analysis are the response variable (percentage of intra-provincial mobility) and the coefficients of variation for the number of hospital beds, nurses, and specialist physicians in each province as the independent variables. Moreover, the fractional regression model indicated that in comparison to the other variables, variation of specialist physicians has the highest and most significant (*P*-value < 0.05) effect on patient mobility. Moreover, with the presence of the latter variable in the model, the effects of the coefficient of variation of beds and the coefficient of variation of nurses are not significant. The results indicated that the imbalance of specialist physicians in each province significantly increases intra-provincial patient mobility, with intra-provincial mobility increasing by 13% with an increase in the specialist coefficient of variation (Table 5).

**Table 5:** Fractional regression model to examine the impact of the coefficients of variation on intra-provincial mobility rate

**Variables**	**Margins**	***P*-Value**
Bed CV	-0.067	0.636
Nurse CV	-0.018	0.884
Specialists CV	0.13	0.009

Due to the significant effect of specialist physician distribution on the intra-provincial mobility rate in Iran, we clustered Iran’s provinces based on the specialist physician coefficient of variation in the secondary analysis of the study. According to the clustering of this variable, six provinces were placed in the first cluster with a low coefficient of variation (lower than 30%), 11 provinces in the second cluster with a medium coefficient of variation (between 30% and 50%), and 14 provinces in the third cluster with a high coefficient of variation (higher than 50%). Table 6 presents the allocation of all provinces to their respective clusters. Cluster analysis showed that the CVs of specialist physicians in the border provinces of Iran, especially in the eastern borders, are higher than other provinces.

**Table 6:** Clustering of provinces based on the specialist coefficient of variation

**Clusters**	**Provinces**	**Ratio of (specialist /population)** **is high**	**Ratio of (specialist /population)** **is moderate**	**Ratio of (specialist /population)** **is low**
**The first cluster** (CV of specialist physicians: **low**)	Tehran, Semnan, Fars, Isfahan, Qom, Sistan and Baluchestan	Tehran, Semnan, Fars, Isfahan,	Qom	Sistan and Baluchestan
**The second cluster** (CV of specialist physicians: **medium**)	Mazandaran, Gilan, Ardabil, West Azerbaijan, Yazd, East Azerbaijan, Kermanshah, Kurdistan, Hamadan, Markazi, Khorasan Razavi	Mazandaran, Gilan, Yazd, East Azerbaijan,	Ardabil, Kurdistan, Markazi, Khorasan Razavi	Hamedan, Kermanshah, West Azerbaijan
**The third cluster** (CV of specialist physician: **high**)	Golestan, Ilam, Khuzestan, Lorestan, Hormozgan, Bushehr, Kohgiluyeh and Boyer Ahmad, Alborz, Qazvin, Zanjan, Chaharmahal and Bakhtiari, Kerman, South Khorasan, North Khorasan	Qazvin	Golestan, Khuzestan, Hormozgan, Bushehr, Kohgiluyeh and Boyer Ahmad, Alborz, Zanjan, Chaharmahal and Bakhtiari, Kerman	Lorestan, Ilam, North Khorasan, South Khorasan

## Discussion

The present study’s findings indicated that the northern and central provinces enjoy better access to resources than the eastern and southern provinces. Moreover, the distribution of specialists, hospital beds, and nurses in Iran’s eastern and southern areas is disproportionate to need. Although remote cities have poor access to health system resources, a significant portion of these few resources is concentrated in the provincial capitals. This is probably why patient mobility in southern and eastern areas of the country is higher than in northern and central areas. Furthermore, southern provinces are located in desert areas with low population density, great distance between cities, and great distance between these areas and the country’s capital. These areas are more deprived than other areas, and in terms of health infrastructure and other welfare infrastructures, they are in worse conditions than other parts of the country. Most doctors and nurses are reluctant to serve in these deprived areas due to low access to amenities and economic and social facilities, so patients have to travel to provincial capital cities to receive curative services [31–33].

Our study showed that the number of specialist physicians and hospital beds in each province has no significant relationship with patient mobility. The specialist physician’s dispersion index (CV) in each province is one of the main determinants of patient mobility. However, Fabbri and Robone’s study showed that the numbers of hospital beds, nurses, and physicians are the most influential factors on patient mobility in Italy [34].

The distribution of health resources is an essential factor in access to resources [35]. Therefore, we added the coefficient of variation of health resources to the model as a resource dispersion variable in different regions of the province. Our results showed that the dispersion status of specialist physicians is more unbalanced than those of hospital beds and nurses. Moreover, the dispersion patterns of hospital beds and nurses are similar due to the close relationship between the distribution of nurses and hospital beds [36]. The fractional regression model indicated that an increase in the number of specialist physicians improves their dispersion and that their equitable distribution within the province is negatively correlated with intra-provincial mobility [37]. Similarly, the results of Saber-Mahani’s study in Iran showed that many patients are willing to leave their cities to receive the services of the doctors they trust and continue their treatment in another city [7].

The present study indicated that the provinces’ human development indices are directly related to patient mobility. In other words, provinces in which people’s literacy, income, and health status were higher had a lower rate of patient mobility. Based on cluster analysis results, it might be said that improving the human development index leads to an improved distribution of specialist physicians in provinces. Previous studies have also shown that most specialists prefer to work in large cities, where people’s economic status is better and there is more access to facilities and amenities [33, 38].

It seems that the high rate of patient mobility in border provinces, especially those along eastern borders, can be associated with the underdevelopment of these regions and the concentration of specialists in provincial capitals. However, the climate in eastern provinces is hot and dry, and the long distance between cities and between cities and capitals make patient mobility significantly more challenging than in other provinces.

Maldistribution of specialists, especially in remote areas, is a global issue [34, 38]. According to Naranong, the lack of medical staff in Thailand’s remote areas and small cities is the main reason for patient mobility [39]. Furthermore, Koylu et al.‘s study results showed a strong relationship between socio-demographic and cultural variables and patient mobility in Turkey [14].

Little research has been conducted on factors influencing patient mobility within a country’s borders so far. Lamonta associates the high geographic mobility of psychiatric patients in London to unsatisfied demands and high expectations of patients in the use of psychiatric services [40]. Brenna claimed that patient mobility between areas to receive hospital care in Italy indicates the necessity of redistributing resources across these areas. This mobility resulted in increased migration costs and the flow of financial resources from southern Italy to central and northern regions [41]. Saber-Mahani et al. have conducted a study in Iran investigating the factors associated with patient mobility. Their findings showed that most of the services provided to migrant patients were also provided in their place of residence. However, the patients stated that lack of trust and the low quality of services provided in local hospitals are the main reasons for their mobility to province capitals [7].

8Free inter-regional patient mobility reduces health system sustainability over time [8]. In Iran, the patient’s freedom to choose a service provider encourages patient mobility. Patient mobility between areas creates challenges for both patient-sending and patient-receiving cities; for instance, in patient-sending cities, patients’ avoidance of local services that are capable of satisfying their needs further weakens these services and reduces bed occupancy rates in these areas. Therefore, policymakers will gradually be dissuaded from improving services in these areas. On the other hand, increased workload due to the high number of unnecessary visits extends waiting lists and reduces the quality of services in patient-receiving cities. Furthermore, crowds and long queues of patients encourage policymakers to develop more resources in larger cities. This vicious cycle gradually pushes the areas far from curative resources further into poverty and leads to the concentration of all resources in metropolitan areas [5, 7].

Countries have implemented different policies to reduce the adverse effects of unnecessary patient mobility. For instance, the Italian healthcare system has increased taxes on treatment expenses for citizens who move to other areas to receive healthcare services [42]. Similarly, people have to pay additional taxes for these kinds of services in Thailand, and the generated income is used to improve doctor training [39].

While the available literature mainly focuses on inter-provincial patient mobility [14, 43, 44], this study analyzed intra-provincial patient mobility and its influencing factors. This analysis is important because, in Iran, at least one state university of medical sciences is responsible for the population’s health in every province. All universities of medical sciences have common goals and functions and operate with the same policies. However, patient mobility varies from province to province. This study provides a new perspective on the factors that affect patient mobility in Iran provinces.

This study showed that the dispersion of specialist physicians influences patient mobility in Iran. This result has good news for health system policymakers because although coping with the dispersion of hospital beds is very costly, time-consuming, and sometimes impossible, promoting specialist distribution is more feasible. Over the past two decades, the specialist training capacity of Iran has more than doubled, and Iran will no longer face a shortage of specialists [41]. However, training more specialists does not lead to equal access to their services if their distribution is not even. Implementing policies to change health human resource distribution is a complicated process that needs to be supported by evidence and legislation. In order to make a list of solutions, best practices in the distribution of specialists in other health systems should be reviewed; study of cases like distribution of physicians in Britain, where the income level is not a barrier to access to physicians, will be beneficial [38].

Patient mobility status is an important factor in estimating the number of hospital beds required. For the first time in a national survey, the study team produced a patient matrix of mobility within the provinces of Iran. The present study is the first to provide a comprehensive analysis of patient mobility status and its determining parameters in Iran. Previously, there was no analysis of the patient mobility status within provinces of Iran. Iran is a vast and populous country, and it seems that the present study results can help analyze factors affecting patient mobility in similar countries of the world.

### Limitations

One of the limitations of this study was the use of one-month patient mobility data. Due to the lack of a patients’ mobility registry system in Iran, we held a data-gathering survey across the country to gather patients’ mobility data, which was done in August. furthermore, patient mobility rate is calculated on a single month, while the independent variables are mainly annual. However, given that we used patient mobility rate in our analysis, not patient admission data, we assumed that the pattern might be the same all year round, because independent variables such as health resource distribution and indicators, don’t change very fast during a year. Akbari Sari et al.‘s study showed that between 2001 and 2011, the annual changes in the distribution of health resources in Iran were less than 5%, so the number of hospital beds, pharmacies, laboratory centers, rehabilitation centers, radiology centers, and health house has been increased by 4.2%, 2.3%, %1%, 5.6%, 2.5%, and 1% respectively [45]. Yet, conducting a study with one-year data will help a lot to more accurately understand the situation of patient movement in Iran.

Since the patient medical information registration system collects data separately in each province, reliable patient movement data is available only in provincial level not national level. As a result, we weren’t able to analyze inter-provincial patients’ mobility. It seems creating a national platform for Iran that facilitates patients’ data exchange between provinces realize many benefits for health system policymakers.

## Conclusion

Although analyzing single-month data may have some limitations, the findings of this study indicated that the distribution of specialist physicians across the country has more impact on patient mobility rates than the distribution of hospital beds and nurses. Therefore, any measure to provide equitable distribution of specialist physicians throughout each province must start from provinces in the third cluster with high specialist coefficients of variation.

The absence of a referral system, patients’ freedom to choose among providers across the country, and citizens’ distrust of and unawareness of services available in their place of residence lead to unnecessary patient mobility, and therefore, cause severe damage to the health system and the society. Authorities and policymakers must adopt policies to control unnecessary patient mobility to protect society’s long-term interests and contribute to the development of equality. These policies must be modeled on the best practices of other communities and be designed and implemented after receiving the views of domestic experts.

## Supplementary Information

**Additional file 1.**

## Data Availability

Regarding to “Data availability”, it should be noted that the research team is ready to provide access to research data for the judges and the audience of the journal. If you need data from this study, you can contact the corresponding author.

## Abbreviations

HIS: hospital information systems
CV: coefficient of variation
PM: patient mobility
HB: number of hospital beds
SP: number of specialist physicians
HDI: human development index
THE: total health expenditure
PD: population density
U5MR: under-five mortality rate
HBCV: coefficient of variation of hospital beds
NCV: coefficient of variation of nurses
SPCV: coefficient of variation of specialist physicians

## Declarations

In this study, the home address data of patients admitted to hospitals as well as information on the distribution of health resources in different regions of the country have been used. Data related to the patient’s mobility were provided collectively without mentioning individual patients names or national ID. As a result, these data are not subject to the conditions set out in the Helsinki Declaration. Given that no human sample participated directly in this study, the informed consent of the participants in this study haven’t been obtained.
