# Supplementary material for: Factors associated with patients’ mobility rates within the provinces of Iran
Source: BMC Health Serv Res. 2022 Dec 20;22:1556. doi: 10.1186/s12913-022-08972-6 (PMC9764717; doi:10.1186/s12913-022-08972-6)
Supplement: Supplementary file 1 — Additional file 1. [file 12913_2022_8972_MOESM1_ESM.docx]

Supplementary file:

In general, the negative binomial regression model is as follows:

$$\ln\left( E(y_{i}|x_{i}) \right)=\beta_{0}+\beta_{1}x_{1}+\beta_{2}x_{2}+\ldots+\beta_{p}x_{p}$$

where

$x_{i}$: independent variables

$\beta_{j}$: regression coefficients

$y_{i}$: response variable (intra-provincial mobility rate)

Generally, the fractional regression model is as follows:

$$E\left( y | x \right)=\beta_{0}+\beta_{1}x_{1}+\beta_{2}x_{2}+\ldots+\beta_{p}x_{p}$$

in which

$x_{i}$: independent variables

$\beta_{j}$ : regression coefficients

$y_{i}$: responding variable (intra-provincial mobility rate)
